# Assessing the development and implementation of the Global Trigger Tool method across a large health system in Sicily

**DOI:** 10.12688/f1000research.18025.4

**Published:** 2020-07-15

**Authors:** Vincenzo Parrinello, Elena Grasso, Giuseppe Saglimbeni, Gabriella Patanè, Alma Scalia, Giuseppe Murolo, Peter Lachman

**Affiliations:** 1 U.O. Qualità e Rischio Clinico, Azienda Ospedaliero-Universitaria “Policlinico-Vittorio Emanuele, Catania, 95129, Italy; 2 Servizio 8 “Qualità, Governo Clinico e Sicurezza del Paziente”, Assessorato della Salute, Regione Siciliana, Palermo, 90145, Italy; 3 International Society for Quality in Healthcare, Dublin, D02NY63, Ireland

**Keywords:** Global Trigger Tool, patient safety, adverse events detections, quality of care, medical errors, harm

## Abstract

**Background:** The Institute for Healthcare Improvement (IHI) has proposed a new method, the Global Trigger Tool (IHI GTT), to detect and monitor adverse events (AEs) and provide information to implement improvement. In 2015, the Sicilian Health System adopted IHI GTT to assess the number, types and severity levels of AEs. The GTT was implemented in 44 of 73 Sicilian public hospitals and 18,008 clinical records (CRs) were examined. Here we present the standardized application of the GTT and the preliminary results of 14,706 reviews of CRs.

**Methods:** IHI GTT was adapted, developed and implemented to the local context. Reviews of CRs were conducted by 199 professionals divided into 71 review teams consisting of three individuals: two of whom had clinical knowledge and expertise, and a physician to authenticate the AE. The reviewers entered data into a dedicated IT-platform. All 44 of the public hospitals were included, with approximately 300,000 yearly inpatient admissions out of a population of approximately 5 million. In total, 14,706 randomized CRs of inpatients from medicine, surgery, obstetric and ICU wards, from June 2015 to June 2018 were reviewed.

**Results:** In 975 (6.6%) CRs at least one AE was found. Approximately 20,000 patients of the 300,000 discharged each year in Sicily have at least one AE. In 5,574 (37.9%) CRs at least one trigger was found. A total of 1,542 AEs were found. The analysis of ROC curve shows that the presence of two triggers in a CR indicates with high probability the presence of an AE. The most frequent type of AE was in-hospital related infection.

**Conclusions:** The GTT is an efficient method to identify AEs and to track improvement of care. The analysis and monitoring of some triggers is important to prevent AEs. However, it is a labor-intensive method, particularly if the CRs are paper-based.

## Introduction

Safety is one of the domains of quality in healthcare. Improving the safety of patients is a political priority worldwide, as studies on the safety of patients have drawn attention to the high rates of health care-related harm ^1– 3^. Improving patient safety requires effective and reliable methods to identify and monitor adverse events (AEs) so that learning can take place and improvements can be made.

Even though several methods to detect AEs are available, there is no universally recognized method that reliably provides a comprehensive overview of the extent of the problem. These methods include incident reporting, clinical records (CRs) review and automated extraction using hospital administrative data, for example Patient Safety Indicators (PSI) as developed by the Agency for Healthcare Research and Quality (AHRQ). Incident reporting is the most commonly used method to detect AEs in hospitals, but is based on voluntary reporting. Despite considerable efforts by local hospitals, reporting systems only detect a limited number of AEs ^4^. The effectiveness of automated extraction using hospital administrative data for detecting AEs depends on the accuracy of data compilation. CRs review, as used in the Harvard Medical Practice Study, is very labor intensive, thereby limiting its use ^4^. As a result, health services, governments and researchers have focused on developing harm detection tools.

This paper is one of the first to report the findings of the application of the Global Trigger Tool, developed by the Institute for Healthcare Improvement (IHI), across the whole health system in Sicily. In 2015, the Sicilian Health System adopted IHI GTT ^5^ to assess the number, types and severity levels of AEs.

## Methods

### IHI GTT

The measurement instrument used in our study is the Italian version of the IHI GTT ^6^. The Italian version was adapted to be appropriate to the regional context ^7^. The triggers are grouped into the same seven categories as the original version (care, medication, surgical, intensive care, obstetric, pediatrics and emergency care); however, changes to some triggers have been introduced. We did not consider triggers and AEs that were present on admission and we added three new triggers: change in procedure anesthesia, duration of surgery greater than 6 hours, and hospital stay greater than five days after delivery ^7^.

### Sample selection

89 of medicine, surgery, obstetrics and intensive care wards has participated in the study. Some of these has participated from June 2015 to June 2018, while others did not continue to the end of the observation period. Others were added during the study. Some departments participated in the study from June 2015 to June 2018, while others did not continue until the end of the observation period. Others were added during the study.

We used a random method of records selection, as recommended by the IHI GTT. In each ward, 10 inpatient CRs were randomly and monthly selected, one every ten. ( Table 1) From the ordered sequence of the numbering of the CRs of the period under evaluation, a CR was selected every 10 (ie 10 ^th^, 20 ^th^, 30 ^th^, 40 ^th^, 50 ^th^, etc). In the case in which the number of patients discharged during that month was less than 100, we proceded to remove the previously selected CRs and to select another CR in the same way (one CR every 10). Eligibility criteria were an admission lasting more than 24 hours, and all the administrative data completed. In the Intensive Care Unit (ICU), all patient CRs, discharged during the reference period, were reviewed.

**Table 1.** Distribution of CRs per clinical area.

	Medicine	Surgery	Obstetric	ICU	Total
CRs examined, n (%)	4571 (31.1)	4826 (32.8)	3336 (22.7)	1973 (13.4)	14706 (100)
CRs with triggers per CRs examined, n (%)	1571 (34.3)	1709 (35.4)	676 (20.2)	1672 (84.7)	5574 (37.9)
CRs with isolated trigger CRs examined, n (%)	930 (20.3)	1085 (22.5)	491 (14.7)	272 (13.7)	2778 (18.9)
CRs with AEs, n (%)	191 (19.5)	128 (14.2)	57 (5.8)	599 (61.5)	975 (100)
AEs, n (%)	210 (13.5)	138 (9.0)	61 (3.9)	1133 (73.5)	1542 (100)
CRs with AEs/CRs examined, (%)	4.2	2.7	1.7	30.4	6.6
CRs with AEs/CRs with triggers, (%)	11.1	8.4	8.4	35.9	17.5

CRs, clinical records; ICU, intensive care unit; AES, adverse events

### Review team

As per the IHI protocol, each review team was composed of three individuals: two with clinical knowledge and expertise on patient clinical documentation, and a physician whose role was to authenticate the findings and the severity rating of the AEs.

The total number of the reviewers was 199 divided into a 71 team. Where possible, the review team remained consistent over time.

### Review process

We excluded triggers and/or events that took place outside the time of the patient admission to the hospital and we considered only triggers and AEs that occurred during hospitalization. The two clinical reviewers audited all the CRs on their own, independently. We used five worksheets: general care, medication, surgical, obstetric and intensive care, with some changes in accordance with the IHI GTT. The third reviewer was always a physician. The physician did not review the CRs, but had to authenticate the consensus of the two primary record reviewers. The physician authenticated the findings of the adverse events, the rating of severity and provided answers to questions of the record reviewers about findings in a specific record. We have used the IHI GTT definition of an adverse event: *unintended physical injury resulting from or contributed to by medical care that requires additional monitoring, treatment or hospitalization, or that results in death.*

Triggers and AEs present at the time of hospitalization were excluded in this study.

The CRs were examined following the order of the sections described in the IHI GTT. The revison time should have been no longer than 20 minutes. The “20-minute rule” was applied to all records regardless their size ^5^. The reviewers entered data into a specially developed dedicated IT-platform, developed by our IT team (based on Jawascript HTML and PHP) ^8^

### Statistical analysis

For the statistical analysis we used the software SPSS ver. 20. We used it also to develop the Receiver Operating Characteristic (ROC) curve analysis.

## Results

From June 2015 to June 2018, 18,008 CRs from 105 wards of 44 Sicilian public hospitals were examined. In this study, we analyzed just 14,706 CRs relating to patients discharged from 89 medicine, surgery, obstetrics and intensive care wards, without including the CRs of the emergency and pediatric wards. In 5,574 (37.9%) CRs at least one trigger in each patient was found. In 7 CRs, the reading of the discharge diagnosis aroused interest by reviewers and an AE was detected and the triggers were not looked for. AEs were determined in 1,542 CRs ( Table 1). The identification of triggers allowed us to identify corresponding AEs ( Table 2).

**Table 2.** Distribution of general care triggers and AEs.

Trigger	Description	Number of times detected, n (%)	Number of times associated, with AEs, n (%)	Trigger	Description	Number of times detected, n (%)	Number of times associated, with AEs, n (%)
**C01**	Blood products use	2002 (26.7)	958 (15.7)	**S01**	Return to surgery	64 (20)	47 (31.8)
**C02**	Emergency and rescue	719 (9.6)	617 (10.1)	**S02-A**	Change in procedure: surgery	57 (18)	8 (5.4)
**C03**	Acute dialysis	279 (3.7)	438 (7.2)	**S02-B**	Change in procedure: anesthesia	6 (1.8)	0 (0)
**C04**	Positive blood culture	291 (3.9)	485 (7.9)	**S03**	Admission to ICU	31 (9.6)	13 (8.8)
**C05**	X-ray or Doppler studies for emboli or DVT	435 (5.8)	112 (1.8)	**S04**	Intubation/reintubation/BiPap in PACU	10 (3.1)	7 (4.7)
**C06**	Decrease of Hb or Ht >25%	1433 (19.1)	896 (14.7)	**S05**	X-ray intraoperative or in PACU	4 (1.2)	0 (0)
**C07**	Patient fall	29 (0.4)	20 (0.3)	**S06**	Intraoperative or postoperative death	9 (2.8)	2 (1.4)
**C08**	Pressure ulcers	254 (3.4)	449 (7.4)	**S07**	Mechanical ventilation >24 hours post-op	18 (5.6)	9 (6.1)
**C09**	Readmission within 30 days	294 (3.9)	115 (1.9)	**S08**	Intraoperative epinephrine, norepinephrine, naloxone, or flumazenil	7 (2.2)	2 (1.4)
**C10**	Restraint use	260 (3.5)	38 (0.6)	**S09**	Postoperative troponin level >1.5 ng/mL	19 (5.9)	3 (2.0)
**C11**	Health care– associated infection	504 (6.7)	894 (14.6)	**S10**	Injury, repair, or removal of organ	13 (4.0)	9 (6.1)
**C12**	In-hospital stroke	35 (0.5)	78 (1.3)	**S11**	Any operative complication	60 (19)	41 (27.7)
**C13**	Transfer to higher level of care	678 (9.0)	595 (9.7)	**S12**	Duration of surgery > 6h	25 (7.7)	7 (4.7)
**C14**	Any procedure complication	284 (3.8)	408 (6.7)	**TOTAL**		**323 (100)**	**148 (100)**
**TOTAL**		**7.497 (100)**	**6.103 (100)**	**P01**	3rd- or 4th-degree lacerations	13 (2.6)	7(10.9)
**M01**	Clostridium difficile– positive stool	28 (1.0)	21 (1.4)	**P02**	Platelet count less than 50,000	2 (0.4)	0 (0)
**M02**	PTT >100 seconds	99 (3.4)	122 (8.0)	**P03**	Estimated blood loss >500 mL (vaginal) or >1000 mL (C-section)	27 (5.5)	13 (20.3)
**M03**	INR > 6	48 (1.7)	33 (2.2)	**P04**	Specialty consult	96 (19.5)	11 (17.2)
**M04**	Glucose < 50 mg/dl	225 (7.8)	238 (15.6)	**P05**	Administrate prostaglandins postpartum	97 (19.7)	8 (12.5)
**M05**	Rising BUN or serum creatinine >2 times baseline	1194 (41.5)	718 (47.1)	**P06**	Instrumented delivery	101 (20.5)	7 (10.9)
**M06**	Vitamin K administration	231 (8.0)	155 (10.2)	**P07**	General anesthesia	75 (15.2)	10 (15.6)
**M07**	Anti-allergic use	145 (5.0)	49 (3.2)	**P08**	Hospital stay> more than 5 days	81 (16.5)	8(12.5)
**M08**	Flumazenil use	37 (1.3)	24 (1.6)	**TOTAL**		**492 (100)**	**64 (100)**
**M09**	Naloxone use	7 (0.2)	6 (0.4)	**I01**	Pneumonia onset	193 (10.6)	380 (19.8)
**M10**	Anti-emetic use	843 (29.3)	133 (8.7)	**I02**	Readmission ICU	59 (3.2)	125 (6.5)
**M11**	Over-sedation	21 (0.7)	26 (1.7)	**I03**	In-unit procedure	761 (41.9)	679 (35.3)
**TOTAL**		**2,878 (100)**	**1,525 (100)**	**I04**	Intubation/reintubation	804 (44.2)	740 (38.5)
				**TOTAL**		**1,817 (100)**	**1,924 (100)**

AEs, adverse events; ICU, Intensive Care Unit; PACU, Post Anesthesia Care; Hb, Hemoglobin; Ht, hematocrit; DVT, deep venous thrombosis; PTT, Partial Thromboplastin Time; INR, International Normalized Ratio; BUN, Blood Urea Nitrogen.

In this study, 37.9% (n=5,574) of all CRs examined had at least 1 positive trigger. Of those, 2,778 CRs had a single positive trigger (49.8% of all CRs with positive triggers) while 2,796 CRs had more than one positive trigger (51.2% of all CRs with positive triggers).

CRs with triggers (n=5,574) are significantly present in surgery wards (n=1,709; 35.4%), medicine wards (n=1,517; 34.3%) and ICU (n=1,672; 84.7%). while CRs with triggers in obstetrics wards are significantly less frequent (n=676; 20.3%). CRs with isolated triggers are more common in medical wards (n=1,085; 23.7%) and rarer in ICU (n=272; 13.7%) ( Table 1).

We detected 1542 AEs in 975 CRs (i.e. patients) with AEs. In 652 patients (66.8%) a single AE was present. In the remaining 323 (33.2%) were more than one AEs.

**Figure 1.**
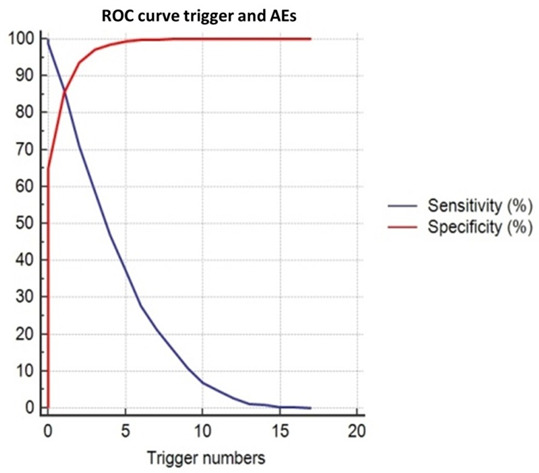
ROC analysis of two random triggers. ROC curve shows that the presence of two triggers in clinical records indicates an adverse event with a high probability.

This analysis allowed us to highlight how isolated triggers are not always a good indicator of AEs. A Receiver Operating Characteristic (ROC) curve analysis demonstrates that the presence of two triggers in a CR has a high probability that an AE having occurred ( Figure 1). In CRs with a high frequency of triggers, a corresponding number of AEs was not always detected. As indicated in Table 3, on the contrary, some triggers were associated with a large number of AEs. For example, the trigger C11 Health care–associated Infection was detected in 504 CRs. However, often, a single CR with trigger C11, presented more than one AE.

**Table 3.** Distribution of triggers and AEs.

Trigger		Triggers with AEs (n)	AEs with trigger (n)
C11	Health care–associated Infection	504	894
C04	Positive blood culture	291	485
C14	Any procedure complication	284	408
C03	Acute dialysis	279	438
C08	Pressure ulcers	254	449
M04	Glucose < 50 mg/dl	225	238
I01	Pneumonia onset	193	380
M02	PTT >100 seconds	99	122
I02	Readmission ICU	59	125
C12	In-hospital stroke	35	78
M11	Over-sedation	21	26

AEs, adverse events; ICU, Intensive Care Unit; PTT, Partial Thromboplastin Time.

Triggers and AEs were analyzed when isolated triggers were identified ( Table 4). For example, the isolated trigger C01 (Blood products use) was present in 483 cases, but identified only two AEs, and the trigger M05 (Rising BUN or serum creatinine >2 times the baseline) did not identify any AEs. AEs were classified using the 2009 edition of the WHO International Classification for Patient Safety (ICPS) ^9^, and a clinical classification developed by our group ( Table 5).

**Table 4.** Distribution of isolated triggers and AEs.

Trigger	Description	number of times detected isolated, n (%)	number of times associated with AEs, n (%)	Trigger	Description	number of times detected isolated, n (%)	number of times associated with AEs, n (%)
**C01**	Blood products use	483 (33.7)	2 (2.5)	**S01**	Return to surgery	12 (15.0)	5 (41.7)
**C02**	Emergency and rescue	91 (6.4)	4 (5.0)	**S02-A**	Change in procedure: surgery	32 (40.0)	1 (8.3)
**C03**	Acute dialysis	6 (0.4)	4 (5.0)	**S02-B**	Change in procedure: anesthesia	3 (3.8)	0 (0)
**C04**	Positive blood culture	26 (1.8)	11 (13.8)	**S03**	Admission to ICU	5 (6.3)	0 (0)
**C05**	X-ray or Doppler studies for emboli or DVT	186 (13.0)	0 (0)	**S04**	Intubation/reintubation/BiPap in PACU	1 (1.3)	0 (0)
**C06**	Decrease of Hb or Ht >25%	190 (13.3)	0 (0)	**S05**	X-ray intraoperative or in PACU	2 (2.5)	0 (0)
**C07**	Patient fall	9 (0.6)	4 (5.0)	**S06**	Intraoperative or postoperative death	1 (1.3)	0 (0)
**C08**	Pressure ulcers	23 (1.6)	15 (18.8)	**S07**	Mechanical ventilation >24 hours post-op	1 (1.3)	0 (0)
**C09**	Readmission within 30 days	114 (8.0)	9 (11.3)	**S08**	Intraoperative epinephrine, norepinephrine, naloxone, or flumazenil	0 (0)	0 (0)
**C10**	Restraint use	115 (8.0)	2 (2.5)	**S09**	Postoperative troponin level >1.5 ng/mL	2 (2.5)	0 (0)
**C11**	Health care–associated infection	32 (2.2)	17 (21.3)	**S10**	Injury, repair, or removal of organ	4 (5.0)	1 (0)
**C12**	In-hospital stroke	3 (0.2)	0 (0)	**S11**	Any operative complication	16 (20.0)	5 (41.7)
**C13**	Transfer to higher level of care	97 (6.8)	1 (1.3)	**S12**	Duration of surgery > 6h	1 (1.3)	0 (0)
**C14**	Any procedure complication	58 (4)	11 (13.8)	**TOTAL**		**80 (100)**	**12 (0)**
**TOTAL**		**1433 (100)**	**80 (100)**	**P01**	3rd- or 4th-degree lacerations	9 (3.3)	4 (36.4)
**M01**	Clostridium difficile–positive stool	7 (0.7)	1 (5.6)	**P02**	Platelet count less than 50,000	1 (0.4)	0 (0)
**M02**	PTT >100 seconds	13 (1.4)	0 (0)	**P03**	Estimated blood loss >500 mL (vaginal) or >1000 mL (C-section)	5 (1.9)	3 (27.3)
**M03**	INR >6	5 (0.5)	0 (0)	**P04**	Specialty consult	52 (19.3)	0 (0)
**M04**	Glucose < 50 mg/dl	53 (5.6)	4 (22.2)	**P05**	Administrate prostaglandins postpartum	48 (17.8)	0 (0)
**M05**	Rising BUN or serum creatinine >2 times baseline	263 (27.7)	0 (0)	**P06**	Instrumented delivery	77 (28.6)	2 (18.2)
**M06**	Vitamin K administration	31 (3.3)	2 (11.1)	**P07**	General anesthesia	35 (13.0)	2 (18.2)
**M07**	Anti-allergic use	60 (6.3)	7 (38.9)	**P08**	Hospital stay > 5 days after delivery	42 (15.6)	0 (0)
**M08**	Flumazenil use	10 (1.1)	0 (0)	**TOTAL**		**269 (100)**	**11 (100)**
**M09**	Naloxone use	1 (0.1)	0 (0)	**I01**	Pneumonia onset	8 (17.4)	0 (0)
**M10**	Anti-emetic use	504 (53.1)	4 (22.2)	**I02**	Readmission ICU	1 (2.2)	0 (0)
**M11**	Over-sedation	3 (0.3)	0 (0)	**I03**	In-unit procedure	15 (32.6)	0 (0)
**TOTAL**		**950 (100)**	**18 (100)**	**I04**	Intubation/reintubation	22 (47.8)	1 (100)
				**TOTAL**		**46(100)**	**1(100)**

AEs, adverse events; ICU, Intensive Care Unit; PACU, Post Anesthesia Care; Hb, Hemoglobin; Ht, hematocrit; DVT, deep venous thrombosis; PTT, Partial Thromboplastin Time; INR, International Normalized Ratio; BUN, Blood Urea Nitrogen.

**Table 5.** Categorization of adverse events.

International Classification for Patient Safety (ICPS) - WHO ed. 2009		Clinical classification	
INCIDENT TYPE	AEs, N (%)	INCIDENT TYPE	AEs, N (%)
Healthcare Associated Infection	742 (48.1)	Healthcare Associated Infection	742 (48.1)
Clinical Process/Procedure	697 (45.2)	Surgical complications	175 (11.3)
Medication/IV Fluids	89 (5.7)	Pressure ulcers	172 (11.2)
Patient Accidents	12 (0.1)	Acute kidney injury	133 (8.6)
Blood/Blood Products	2 (0.1)	Procedure complications *	109 (7.1)
TOTAL	1542 (100)	Hypoglycemia	62 (4.0)
		Delivery complications	47 (3.0)
		In-hospital Stroke	18 (1.2)
		Anesthetic complications	17 (1.1)
		Hemorrhage	5 (0.3)
		Various	62 (4.0)
		TOTAL	1542 (100)

AEs, adverse events. * Endoscopic procedures, central catheterization, urinary catheterization, orotracheal intubation.

The most frequent type of AEs observed: in-hospital related infections; surgical complications; pressure ulcers; acute kidney injury; and procedure complications.

## Discussion

The evaluation of the quality and safety of health systems is difficult, but has become a priority of healthcare funders and organizations. Outcome, management and patient satisfaction indicators are available to measure the different dimensions of health care quality, but reliable measurements of safety have been elusive. Many methodologies and indicators, such as the PSI developed by AHRQ, the review of health documentation incident reporting and prospective clinical surveillance methodology are currently used.

The documentation and study of AEs, i.e. where they occur, and the type and degree of harm, is essential to promote specific opportunities for interventions improvement and to evaluate effectiveness of any intervention over time.

The IHI GTT is one methodology proposed to detect and monitor AEs and provide information to implement improvement. At present, compared to other methods, it may be the best methodology to use ^4^. A systematic review reported the use of GTT methodology in 15 countries in 44 hospitals, with 79,004 clinical records examined ^10^. The data are an underestimation, as the report did not include some comprehensive Swedish and Norwegian studies ^11^. Recently, papers have been published in Italy, Austria, China and Russia ^12– 16^. A critical appraisal of the studies and their results is difficult, as the methodologies use are heterogeneous, protocols are often locally adapted to the local context, the populations studied are different, and the skills of the reviewers vary. We adapted the IHI GTT to the local context in Sicily for this study and did not consider triggers and AEs identified at the admission of the patient as well as modifying some triggers. In this study, the triggers were analyzed both when associated with other triggers and when isolated. In both cases the correlation with AEs was analyzed.

### Rates of triggers

In this study, 37.9% (n=5,574) of all CRs examined had at least 1 positive trigger. Of those, 2,778 CRs had a single, positive trigger (49.8% of all CRs with positive triggers) while 2,796 CRs had more than one positive trigger (51.2% of all CRs with positive triggers).

The connection between the number of CRs with triggers and the number of CRs examined is not always reported in the literature and when reported it is not always clear. Xu *et al.* ^17^ report that during review of 240 clinical records, 51.0% triggers (26/51) were identified 206 times. Mortaro *et al.* ^12^ report that during review of 1,320 clinical records, a total of 130 triggers were detected.

In our study, few AEs were identified by isolated triggers and many isolated triggers are not associated with AEs. Not very useful for identifying AEs, some triggers may be direct measures of "near misses". Today's trigger could be tomorrow's adverse event.

In general, it would appear that little attention is paid to triggers if they are not related to an AE. Instead, many triggers of the IHI GTT protocol could be considered to be a measure of near misses and potential AEs. These include, decrease of Hb or Ht >25%, readmission within 30 days, transfer to higher level of care, clostridium difficile–positive stool, PTT >100 seconds, INR > 6, glucose < 50 mg/dl, rising BUN or serum creatinine >2 times baseline, blood loss >500 mL (after vaginal delivery) or >1000 mL (Cesarean section), and readmission to ICU.

### Rates of AEs

In a systematic review, de Vriess *et al.* ^18^ reported that in 8 studies that included 74,485 CRs, the median overall incidence of in-hospital AEs was 9.2%. Another systematic review reported 44 hospitals with 79,004 CRs, had an incidence between 7 and 51% ^10^. In the Sicilian public hospitals, 1,542 AEs were detected in 975 clinical records, corresponding to an incidence of 6.6% of CRs examined, and to 17.5% of CRs with triggers.

The results of this study are not comparable with other studies due to the diversity of detection protocols. This is also demonstrated by the wide frequency variability of AEs reported in the literature (7–51%) ^10^.

In this study the most significant difference, compared to the other studies, is the exclusion of triggers and AEs present at the time of the patient admission in the hospital.

ICUs have the highest incidence of AEs, both with respect to CRs examined (30.4%) and those with triggers (35.9%) ( Table 1). This could be due to patients being transferred to ICU and the cause of the AE was in another clinical setting. We analyzed the AEs comparing them to the triggers to allow for their identification:

￭ Most AEs are associated with general care triggers (from C1 to C14) (n=6,103) ( Table 2). If triggers are isolated, AEs are more frequently associated with care triggers (n=80) ( Table 4).
￭ The triggers related to general care have been identified 7,497 times, with an AE in 6,103 (81.4%).
￭ Medications-related triggers (From M1 to M11) have been identified 2,878 times, with an AE in 1,525 (53%).
￭ Surgery-related triggers (form S1 to S12) have been identified 323 times, with an AE in 148 (45.8%).
￭ Obstetrics-related triggers (from P1 to P8) have been identified 492 times, with an AE in 61 (12.4%).
￭ Intensive-care-related triggers (from I1 to I4) have been identified 1,817 times with an AE in 1,924 (i.e. it is very common for triggers to identify more AEs in the same patient) ( Table 1 and Table 2).

However, if isolated triggers are considered, the intensive-care-related triggers were detected 46 times and they were correlated with only one AE ( Table 4). This observation suggests that isolated triggers rarely allow to identify an AE and that the strength of the IHI’s GTT methodology is linked to the association of triggers. It is evident that the detection of many triggers in a CR is associated with a high probability of AEs. The analysis of the ROC curve ( Figure 1) shows that it is sufficient to detect two triggers in a CR because it can be almost certain that in that CR there may be an AE.

We have classified the AEs using the ICPS 2009 classification and a clinical classification, developed by our group. In both classifications, hospital acquired infections are the most frequent AEs present ( Table 5), observed in the ICU in 625 clinical records (84.3%). Surgical complications (n=175) were observed in 55.9% (n=99) in ICU, i.e. they were AEs in patients undergoing surgery and then transferred to ICU due to the onset of a complication. In 44.6% (n=80), the AEs are represented by hemorrhagic complications (intra- and post-operative hemorrhages or hematomas). Pressure ulcer lesions were detected in 172 cases, usually in the ICUs (n=112 - 65.1%). Also, the complications from procedures (n=109) were observed mainly in the ICU (n=78; 71.5%). In total, 33 complications from procedures are related to orotracheal intubation, 22 at central venous catheter, and 10 at childbirth analgesia. The complications of child birth (n= 47) were represented more frequently in 59.5% (n=28) by bleeding and in 29.8% (n=14) by lacerations.

### Limitations

Our study has some several limitations. The first concerns the inter-rater reliability assessment of review teams that is not available. The second limitation is the underlying quality of CRs, which may have affected the results. However, all reviewers received the same training and each team followed the same protocols to ensure reliability. Another limitation was the limited number of hospital wards that were included in this study.

## Conclusion

The Global Trigger Tool is an effective method to identify the risk of AEs and track improvement of care. It provides to clinical teams an understanding of the patient safety issues that are present in their clinical area, as well as opportunities to improve. With active involvement of clinical teams, it places patient safety in the centre of clinical activity and fosters a culture of safety. It also provides an effective way to assess the quality of the clinical records. The drawback is that the process is labor intensive, particularly if the clinical records are paper based. The introduction of electronic medical records would allow a quicker process with the automation of the identification of triggers and the possibility to link triggers together in the identification of adverse events and near misses, especially where there has been more than one trigger ^19, 20^. Finally, we conclude that the analysis and monitoring of some triggers, as potential indicators of near misses, is important to prevent adverse events. Today's trigger could be tomorrow's adverse event.

## Ethical considerations

Since the data used in this study was gathered during routine practice and is used for analysis of hospital procedures, no ethical approval was obtained. Every patient gave written informed consent on admission to hospital for the use of their data for scientific research. This consent is the "Information on the processing of personal data" with reference to the Italian law n. 196/2003 and Regulation (EU) 2016/679 of the European Parliament and of the Council of 27 April 2016 (UE).

## Data availability

### Underlying data

Harvard Dataverse: Replication data for Developing and implementing the Global Trigger Tool methodology across a large health system in Sicily, https://doi.org/10.7910/DVN/YQNKCC ^21^

Data are available under the terms of the Creative Commons Zero "No rights reserved" data waiver (CC0 1.0 Public domain dedication).
