# Supplementary material for: Assessing the development and implementation of the Global Trigger Tool method across a large health system in Sicily
Source: F1000Res. 2020 Jul 15;8:263. Originally published 2019 Mar 7. [Version 4] doi: 10.12688/f1000research.18025.4 (PMC7308947; doi:10.12688/f1000research.18025.4)
Supplement: Peer review history

# Peer review: Reviewer response for version 3

**DOI:** 10.5256/f1000research.22986.r65023

**Published:** 2020-06-17

**Authors:** Doncho Donev

**Affiliations:** 1 Institute of Social Medicine, Faculty of Medicine, Ss. Cyril and Methodius University of Skopje, Skopje, Macedonia

The paper presents an innovative method for detecting and monitoring triggers for adverse events (AEs) and serious adverse events (SAEs) in a systematic and comprehensive way. With some corrections and additions (Minor revision) the paper might be approved for indexing.

 General comments:

- The title of the manuscript reflects its content, and the clarity of the text of the paper, in general, is satisfactory for such a complex and broad issue related to the quality of health care and diverse triggers for possible AEs and life-threatening SAEs, with a few remarks and suggestions listed below.

Specific comments:

- Abstract: The third sentence from the Abstract-Background (or Introduction and aim) to be moved to the Abstract-Methods.
- The Abstract-Methods, last sentence, the word “medicine” needs to be specified if it means “Internal medicine ward” or broader, including other hospital nonsurgical wards. The same applies in the text of the paper, in Methods-Sample selection.
- The first sentence of the Abstract-Results to start with “In each of 975…”, as well as at the end of the second sentence, after the words “…have at least one AE” to be added “in each patient”.
- Text of the paper: Section Methods-Sample selection, the fifth sentence, after the first words “In each ward”, to continue with “10 inpatient CRs were randomly selected…”:
- Section Results, third sentence, after the words “In 5,574 (37,9%) CRs at least one trigger” to be added “in each patient was found”.
- Table 2, the title, after the words “Distribution of” to be added “general care…”.
- There are too many abbreviations in the tables and in the text and it might be appropriate to prepare a list with explanation at the end of the text or below each table.
- Figure 1, the title, the acronym EAs to be replaced with AEs.
- Table 4, the title, after the word “isolated” to be added word “care”.
- Limitations section, to explain that CRs from limited number of hospital wards were included in the study.
- Conclusion section, the first sentence, after the words “effective method to identify”, to be added “the risk for AEs…”, and the last sentence to start with the words “Without increasing awareness and prevention by implementing clinical protocols, standards of care and Check lists recommended by the World Health Organization…”.
- It’s not necessary to connect references to the text of the Conclusion section.
- References list consist of 21 references connected to the text properly in order of the first appearance. The list might be supplemented with additional references, especially from the most recent years.

Is the work clearly and accurately presented and does it cite the current literature?

Partly

If applicable, is the statistical analysis and its interpretation appropriate?

Yes

Are all the source data underlying the results available to ensure full reproducibility?

Yes

Is the study design appropriate and is the work technically sound?

Yes

Are the conclusions drawn adequately supported by the results?

Partly

Are sufficient details of methods and analysis provided to allow replication by others?

Yes

Reviewer Expertise:

I’m doctor specialist in Social medicine and organization of health care services and professor emeritus in Social Medicine and Public Health from the Faculty of Medicine in Skopje.

I confirm that I have read this submission and believe that I have an appropriate level of expertise to confirm that it is of an acceptable scientific standard.

# Response

**Published:** 2020-06-18

**Authors:** vincenzo parrinello

**Affiliations:** U.O. Quality management and patients safety - Azienda Ospedaliero-Universitaria Catania, Italy, Italy

Dear Professor Donev, thank for your review and your suggestions. We verified and adjusted some typos and added more clarification as you suggested. Thank you for your time and consideration.

Best regards,

Dr. Vincenzo Parrinello

# Peer review: Reviewer response for version 3

**DOI:** 10.5256/f1000research.22986.r54873

**Published:** 2019-10-09

**Authors:** James M. Naessens

**Affiliations:** 1 Division of Health Care Policy and Research, Mayo Clinic, Rochester, MN, USA

Revisions look good.

Is the work clearly and accurately presented and does it cite the current literature?

Yes

If applicable, is the statistical analysis and its interpretation appropriate?

Partly

Are all the source data underlying the results available to ensure full reproducibility?

No source data required

Is the study design appropriate and is the work technically sound?

Partly

Are the conclusions drawn adequately supported by the results?

Partly

Are sufficient details of methods and analysis provided to allow replication by others?

Partly

Reviewer Expertise:

Health Services Research, Patient Safety and Quality of Care Measurement, Biostatistics

I confirm that I have read this submission and believe that I have an appropriate level of expertise to confirm that it is of an acceptable scientific standard.

# Peer review: Reviewer response for version 2

**DOI:** 10.5256/f1000research.22544.r53740

**Published:** 2019-09-19

**Authors:** James M. Naessens

**Affiliations:** 1 Division of Health Care Policy and Research, Mayo Clinic, Rochester, MN, USA

I appreciate the revisions and responses that the authors have made. I believe that the manuscript is now more understandable. I have several editorial suggestions:

- The heading for the fourth column of Table 2 is still confusing. Can you change it to match the heading for the 4th column of Table 3? "AEs with trigger"?
- Delete the duplicate sentence under Sample Selection.
- Change 1st paragraph of Review Process as follows: "The third reviewer was always a physician. The physician did not review the CRs, but had to authenticate the consensus of the two primary record reviewers. The physician authenticated the findings of the adverse events, the rating of severity and provided answers to questions of the record reviewers about findings in a specific record."
- I'm not sure all readers would understand that an "isolated trigger" in Table 1 is a "single positive trigger" in the 2nd paragraph of results. I'd change the sentence to "single, positive trigger".
- Some of the numbers in the 3rd paragraph of Results do not match Table 1. 
- First sentence of 4th paragraph of Results should read: "We detected 1542 AEs in 975 CRs (i.e. patients) with AEs." 
- Finally, under Limitations, first sentence, delete "some". 

Is the work clearly and accurately presented and does it cite the current literature?

Yes

If applicable, is the statistical analysis and its interpretation appropriate?

Partly

Are all the source data underlying the results available to ensure full reproducibility?

No source data required

Is the study design appropriate and is the work technically sound?

Partly

Are the conclusions drawn adequately supported by the results?

Partly

Are sufficient details of methods and analysis provided to allow replication by others?

Partly

Reviewer Expertise:

Health Services Research, Patient Safety and Quality of Care Measurement, Biostatistics

I confirm that I have read this submission and believe that I have an appropriate level of expertise to confirm that it is of an acceptable scientific standard.

# Peer review: Reviewer response for version 1

**DOI:** 10.5256/f1000research.19713.r46773

**Published:** 2019-05-01

**Authors:** Brent C. James

**Affiliations:** 1 Department of Medicine, Stanford University School of Medicine, Stanford, CA, USA

**General comments:**

 The Harvard Medical Practice Study (HMPS – cited by the authors) used post-discharge chart review to detect care-associated injuries (adverse events – AEs) that occurred during hospitalization. Under HMPS, trained nurses reviewed a random selection of charts. If those nurses discovered what they judged to be care-associated injuries, they flagged those events in the chart as a potential AE. Charts that contained one or more AEs were forwarded to 2 independent physician reviewers. If both physician reviewers judged, for each AE, that event had occurred, then it was reported as a confirmed AE. Those physicians also judged whether each AE was avoidable, and whether each could be considered negligent (substandard) care.

 The IHI Global Trigger Tool (IHI GTT) built on the HMPS methodology. It attempted to improve the ability of initial nurse reviewers to detect potential AEs, by providing a set of 51 “review triggers,” falling into 7 major subcategories – explicit initial events that, if detected in the chart, chained to examination of other specific events. Initial assessments of the IHI GTT showed much higher detection rates for AEs than did the original HMPS methodology, which itself far exceeded typical voluntary reporting mechanisms.

 This study reviews the use of the IHI GTT in 44 Sicilian public hospitals across a 3 year time period – June 2015, through June 2018. The study’s authors adapted the IHI GTT to their specific environment. They also added 3 additional review triggers, beyond those included in the original IHI GTT. They focused their analysis on a subset of all charts assessed with their modified IHI GTT: They analyzed only charts for patients hospitalized on medicine, surgery, obstetrics, and intensive care wards.

 As this study notes, many other groups are using the IHI GTT to detect AEs. This work is useful because it supplies empiric observation of one such use, across a large system of hospitals and an extended period of time.

 This report has at least 2 major differences from other work in the field. First, only 6.6% of records reviewed had at least one care-associated adverse event. Other studies have shown much higher AE rates. The authors note this in their text, and suggest it may reflect differences in local environments and chart review methods. Second, most other IHI GTT reviews show adverse drug events (medication-related events - overdoses, drug-drug interactions, and allergic or idiosyncratic reactions) as the dominant category of AEs detected. In this study, they are a distant number 3. Why?

**Specific suggestions:**

1. It appears that the entire IHI GTT program assessed a total of 18,008 records from among 105 hospital wards from June 2015 through June 2018. However, this analysis examines only medicine, surgery, obstetrics, and intensive care wards. Those represented a total 89 wards, with a total of 14,706 records reviewed.
  This is unclear in both your abstract and your text. It would be very helpful to more clearly explain how you derived the CRs included in your analysis.
2. You mention that you had a total of 199 individuals who participated in 71 3-person review teams. Obviously, some people participated in more than 1 team. It is not clear how the 2 initial reviewers shared their work, before their findings were submitted to a physician for final validation. Did they both separately review all records? Did they divide their assigned records between them? Please clarify.
3. In your introduction, 2 ^nd^ paragraph, you review alternative methods for detecting AEs, including incident reporting, CR review, and automated administrative data review (e.g., PSIs). Consider adding “prospective (concurrent) clinical trigger systems,” that track possible real-time clinical responses to AEs then track back to see if an AE actually occurred. Dr. R. Scott Evans at LDS Hospital in Salt Lake City, Utah; and Dr. David Bates at Brigham & Women’s Hospital in Boston, Massachusetts, developed and demonstrated such systems. Such approaches find AEs that never make their way into a traditional clinical record, and may detect far more events.
4. Under Methods, please change the heading “Sampling selection” to say something like “Sample selection.” More importantly, the frame within which “10 inpatient CRs” were selected monthly is not clear. Working the total number of charts reviewed backward, you were probably sampling “10 inpatient CRs” each month for each of the 44 Sicilian public hospitals. Please clarify. How was record selection balanced across the types of wards (medicine, surgery, obstetrics, and ICUs) in each hospital?
5. Under Results, please add clarity regarding the number of patients that had at least 1 AE during their index hospitalization (975); then break out the number of patients who experienced a single AE versus those who had more than 1 AE during their index hospitalization.
6. Table 1: Please clarify – I can’t understand what you mean by the phrases “CRs with triggers per inpatient wards” versus “CRs with trigger isolated per wards”; or “CRs with trigger” versus “CRs with trigger isolated”.
7. Consider moving the first 2 paragraphs of the section entitled “Rates of triggers” from the Discussion section into the Results section.
8. Clarify your text in the first paragraph of your “Rates of triggers” section. For example, it might read:
  “In this study, 37.9% (n=5,574) of all CRs examined had at least 1 positive trigger. Of those, 2,778 CRs had a single positive trigger (49.8% of all CRs with positive triggers) while 2,796 CRs had more than one positive trigger (51.2% of all CRs with positive triggers).
9. Consider modifying Table 2:
  a) Label each of the subsections in Table 2. For example, in your text you cite the Table relative to “general care triggers”. What are “general care triggers”? Presumably you mean the subset of triggers with “C” labels. It would be quite helpful if you put headings on each of the subsections (for C, M, S, P, and I) that labelled the contents of each subsection.
  b) You are (quite appropriately) approaching AE detection as a screening test with 2 main steps. Step 1 involves evaluation of the initial triggers. Step 2 involves following up on positive initial triggers, to see if a validated AE emerges. This is done in the context of a general review by a trained clinical expert, who can (and sometimes does) detect AEs that fall outside of the trigger system.
  It would be very useful if you separated the total time spent on CR review into (a) initial assessment of the triggers; versus follow-up analysis of the positive triggers.
  It would similarly be very useful if, in the 3 ^rd^ column of Table 2 (labelled “Times associated with AEs, n (%)” you showed the proportion of positive triggers that yielded a confirmed AE related to/deriving from the initial positive trigger. The Table, as currently constructed, is quite confusing – in 10 instances (C03, C04, C08, C11, C12, C14, M02, M04, I01, I03) the count in 3 ^rd^ column is larger than the count in 2 ^nd^ column. Presumably, this is because you are not connecting the trigger to derivative AEs (but I can’t really tell, with any certainty).
10. The section entitled “Rates of AEs”, first paragraph, contains the sentence:
  “It would appear that the percentage of clinical records with triggers increases with the increase of percentage of CRs examined, compared to patients discharged, while the percentage of CRs with AEs remains more stable.”
  I can’t translate quite what that means. Please simplify and clarify. It may relate to the 2 upper lines in Figure 2, which both appear to be increasing over time. However, you supply no statistical analysis to show that trends over time in the 2 lines are statistically associated, and you offer no reasoning as to why such a relationship may have meaning.
11. Table 3 appears to identify high frequency triggers, then count the number of AEs of any sort that occur in CRs that had a high frequency trigger. It would be much more useful if you tracked each trigger to related or derivative AEs, rather than treating AEs generically.
12. In the section labelled “Rates of AEs” you have a bulleted list. The last item in that list says:
  “Intensive-care-related triggers have been identified 1,817 times with an AE in 1,924 (i.e., it is very common for triggers to identify more AEs in the same patient)”.
  I think I get the gist of what you mean, but (a) this sentence needs more clarity; and (b) the main purpose for identifying AEs is to move toward intervention and prevention (better, safer care). In that circumstance, treating AEs as generic items is not very useful. It is necessary to list them by specific causes. In that framing, associating triggers (which are cause specific) with a total generic AE count is not very informative or useful. You might want to rethink your framing.

 

**Spelling corrections:**

1. In the Abstract, Methods subsection, it should say “300,000 inpatient yearly admission **s**”.
2. In the Methods section, it should say “Sample selection,” not “Sampling selection”.
3. In the Discussion section, 2 ^nd^ paragraph, it should say “A critical appraisal of the studies and their results is difficult, as the methodolog **ies** use **are** heterogeneous,”.
4. In Figure 1, the label for the blue line should say “ **Sensitivity (%)**,” not “Sensibility (%)”.
5. In the section labelled “Rates of AEs” there is a bulleted list. The 4 ^th^ bullet should say “Surgery- **r**elated” (not “Surgery-elated”).

Is the work clearly and accurately presented and does it cite the current literature?

Yes

If applicable, is the statistical analysis and its interpretation appropriate?

Partly

Are all the source data underlying the results available to ensure full reproducibility?

No source data required

Is the study design appropriate and is the work technically sound?

Yes

Are the conclusions drawn adequately supported by the results?

Partly

Are sufficient details of methods and analysis provided to allow replication by others?

Partly

Reviewer Expertise:

Clinical quality improvement; patient safety; high-reliability organizations

I confirm that I have read this submission and believe that I have an appropriate level of expertise to confirm that it is of an acceptable scientific standard, however I have significant reservations, as outlined above.

# Response

**Published:** 2019-05-28

**Authors:** vincenzo parrinello

**Affiliations:** U.O. Quality management and patients safety - Azienda Ospedaliero-Universitaria Catania, Italy, Italy

We appreciate the review by Professor Btrent of our paper, the accuracy of his observations and the relevance of his suggestions. We are honored that he considers our paper “ *useful because it supplies empiric observation of one such use, across a large system of hospitals and an extended period of time.*”

 We are aware that some of the points require further clarity, which may have been lost as we limited the word count.

**Comment**

*The Harvard Medical Practice Study (HMPS – cited by the authors) used post-discharge chart review to detect care-associated injuries (adverse events – AEs) that occurred during hospitalization. Under HMPS, trained nurses reviewed a random selection of charts. If those nurses discovered what they judged to be care-associated injuries, they flagged those events in the chart as a potential AE. Charts that contained one or more AEs were forwarded to 2 independent physician reviewers. If both physician reviewers judged, for each AE, that event had occurred, then it was reported as a confirmed AE. Those physicians also judged whether each AE was avoidable, and whether each could be considered negligent (substandard) care.*

*The IHI Global Trigger Tool (IHI GTT) built on the HMPS methodology. It attempted to improve the ability of initial nurse reviewers to detect potential AEs, by providing a set of 51 “review triggers,” falling into 7 major subcategories – explicit initial events that, if detected in the chart, chained to examination of other specific events. Initial assessments of the IHI GTT showed much higher detection rates for AEs than did the original HMPS methodology, which itself far exceeded typical voluntary reporting mechanisms.*

**Response**

 Harvard's medical practice study is a key pillar in patient safety and care research. We agree that the IHI Global trigger tool is an attempt to improve the Harvard Medical Practice Study methodology.

 In our opinion, the IHI Global Trigger Tool has two significant differences from the Harvard medical practice study.

 The first is that the IHI Global Trigger Tool is conducted on a random sample of clinical records that are analyzed only if triggers are present.

 The second is that the IHI Global Trigger Tool "focuses on only those adverse events related to the active delivery of care (commission) and excludes, as much as possible, issues related to substandard care” (omission). (IHI Global Trigger Tool for Measuring Adverse Events. Second Edition 2009. http://www.ihi.org/resources/Pages/Tools/IHIGlobalTriggerToolforMeasuringAEs.aspx)

**Comment**

*This report has at least 2 major differences from other work in the field. First, only 6.6% of records reviewed had at least one care-associated adverse event. Other studies have shown much higher AE rates. The authors note this in their text, and suggest it may reflect differences in local environments and chart review methods.*

**Response**

 The percentages of adverse events found in other studies are very different. In a recent systematic review of the literature, the percentage of adverse events is between 7% and 51%. (Hibbert PD, Molloy CJ, Hooper TD, et al .: The application of the Global Trigger Tool: a systematic review. Int J Qual Health Care. 2016; 28 (6): 640-649.) We think that the most important reasons, which can explain these wide differences, could be twofold.

 The first could be due to the different care settings (medicine, surgery, obstetrics, ICU) and therefore to the different complexity of the patients studied. A risk adjustment system would be needed to evaluate the results homogeneously. In our experience, for example, there are significant differences in the AEs rate between apparently homogeneous wards belonging to different hospitals. We have not included these tables to be brief. Since it seems to us that this is very interesting, this aspect will be the subject of other publications.

 The second reason could be due to changes in the detection protocol. The survey methods are, in fact, different in the different studies. We, for example, have turned our attention only to the triggers observed during admission and not to the triggers present at the time of admission. Therefore the adverse events found do not include those present at the time of admission. This could justify the differences in EAs observed compared to other studies.

**Comment**

*Second, most other IHI GTT reviews show adverse drug events (medication-related events - overdoses, drug-drug interactions, and allergic or idiosyncratic reactions) as the dominant category of AEs detected. In this study, they are a distant number 3. Why?*

**Response**

 We do not know why the drug-related AEs observed in our study are infrequent compared to other studies.

 In our study the most frequently detected AEs are Health care–associated Infection. In addition, Rutberg et al. Report that                Health care–associated Infection accounted for 39% of Aes. (Rutberg H, Borgstedt Risberg M, Sjödahl R, et al. Characterization of adverse events detected in a university hospital: a 4-year study using the Global Trigger Tool method. BMJ Open. 2014; 4: e004879.)

 Von Plessen and Coll report that Infections, pressure ulcers procedures-related and gastrointestinal problems were common. (Von Plessen C, Kodal AM, Anhøj J. Experiences with Global Trigger Tool reviews in five Danish hospitals: an implementation study. BMJ Open. 2012; 2: e001324.

 Schildmeijer et al report that " *Overall, the level of agreement for detecting AEs is the level of harm for healthcare-related infections, that is, pneumonia, sepsis and urinary tract infection*." (Schildmeijer K, Nilsson L, Årestedt K, et al. Assessment of adverse events in medical care: lack of consistency between experienced teams using the global trigger tool. BMJ Qual Saf. 2012; 21: 307-314.)

 Landrigan et al. report that " *Harms that were detected in procedures* (186/588)" (Landrigan CP, Parry GJ, Bones CB, et al. Temporal trends in rates of patient harm resulting from medical care. N Engl J Med. 2010; 363: 2124-2134.)

 Mortaro et al. report that the most common types of AEs detected were related to surgical procedures (Mortaro A, Moretti F, Pascu D, et al .: Adverse Events Detection Through Global Trigger Tool Methodology: Results From a 5-Year Study in an Italian Hospital and Opportunities to Improve Interrater Reliability. J Patient Saf. 2017.)

**Comment**

*It appears that the entire IHI GTT program assessed a total of 18,008 records from among 105 hospital wards from June 2015 through June 2018. However, this analysis examines only medicine, surgery, obstetrics, and intensive care wards. Those represented a total 89 wards, with a total of 14,706 records reviewed. This is unclear in both your abstract and your text. It would be very helpful to more clearly explain how you derived the CRs included in your analysis.*

**Response**

 We agree that this may be unclear. From June 2015 to June 2018, 18.008 CRs were examined. In this study, we analyzed 14,706 CRs relating to patients discharged from 89 medicines, surgery, obstetrics and intensive care wards. 16 wards and 3,302 CRs concerned pediatrics wards and emergency department.

**Comment**

*You mention that you had a total of 199 individuals who participated in 71 3-person review teams. Obviously, some people participated in more than 1 team. It is not clear how the 2 initial reviewers shared their work, before their findings were submitted to a physician for final validation. Did they both separately review all records? Did they divide their assigned records between them? Please clarify.*

**Response**

 The total number of reviewers was 199 divided into 71 teams. In some teams, medical reviewers  from another team were the supervisors. The two primary record reviewers should each review all records independently.  The third reviewer was always a physician. The physician, who did not review the records, authenticated the consensus of the two primary record reviewers.

**Comment**

*In your introduction, 2nd paragraph, you review alternative methods for detecting AEs, including incident reporting, CR review, and automated administrative data review (e.g., PSIs). Consider adding “prospective (concurrent) clinical trigger systems,” that track possible real-time clinical responses to AEs then track back to see if an AE actually occurred. Dr. R. Scott Evans at LDS Hospital in Salt Lake City, Utah; and Dr. David Bates at Brigham & Women’s Hospital in Boston, Massachusetts, developed and demonstrated such systems. Such approaches find AEs that never make their way into a traditional clinical record, and may detect far more events.*

**Response**

 This is very interesting.

 In part, this concept is reported in the conclusions *"The introduction of electronic medical records would allow a quicker process with the automation of the identification of triggers and the possibility of linking together in the identification of adverse events and near misses, especially where there has been more than one trigger "*

 We will review the work and attempt to explain this concept better.

**Comment**

*Under Methods, please change the heading “Sampling selection” to say something like “Sample selection.” More importantly, the frame within which “10 inpatient CRs” were selected monthly is not clear. Working the total number of charts reviewed backward, you were probably sampling “10 inpatient CRs” each month for each of the 44 Sicilian public hospitals. Please clarify. How was record selection balanced across the types of wards (medicine, surgery, obstetrics, and ICUs) in each hospital?*

**Response**

 We will change the heading "Sampling selection" to "Sample selection."

 We randomly selected 10 CRs for each department that participated, not for each hospital. The wards that participated were not homogeneously represented in the different hospitals. In some hospitals only the ICU CRs were analyzed. In some hospitals there were no medical, surgical or obstetrics or ICU wards. In others there were more than one medicine, surgery, obstetrics and ICU wards. In addition, hospitals were recruited at different times.

 The CRs sample is therefore not representative. The aim of our study was not to provide a true representation of the patient population present in hospitals during the observation period. Coherently with as reported with the IHI GTT protocol, the main purpose of applying GTT methodology is to produce a sampling approach that is sufficient for the design of safety work in the hospital.

**Comment**

*Under Results, please add clarity regarding the number of patients that had at least 1 AE during their index hospitalization (975); then break out the number of patients who experienced a single AE versus those who had more than 1 AE during their index hospitalization.*

**Response**

 We will indicate the number of patients who experienced a single AE versus those who had more than 1 AE during their index hospitalization.

**Comment**

*Table 1: Please clarify – I can’t understand what you mean by the phrases “CRs with triggers per inpatient wards” versus “CRs with trigger isolated per wards”; or “CRs with trigger” versus “CRs with trigger isolated”.*

**Response**

 We will modify the table.

- The second line "CRs with triggers for inpatient wards" indicates the number and percentage of CRs with one or more triggers.
- The third line "CRs with trigger isolated for wards" indicates the number and percentage of CRs with only triggers.
- The fourth and fifth lines are repetitions. It it is a printing error.

 

**Comment**

*Consider moving the first 2 paragraphs of the section entitled “Rates of triggers” from the Discussion section into the Results section.*

**Response**

 We will move the first 2 paragraphs of the section entitled "Rates of triggers" from the Discussion section into the Results section.

**Comment**

*Clarify your text in the first paragraph of your “Rates of triggers” section. For example, it might read:*

*“In this study, 37.9% (n=5,574) of all CRs examined had at least 1 positive trigger. Of those, 2,778 CRs had a single positive trigger (49.8% of all CRs with positive triggers) while 2,796 CRs had more than one positive trigger (51.2% of all CRs with positive triggers).*

**Response**

 Thank you for this good advice. We will modify accordingly.

**Comment**

*a) Label each of the subsections in Table 2. For example, in your text you cite the Table relative to “general care triggers”. What are “general care triggers”? Presumably you mean the subset of triggers with “C” labels. It would be quite helpful if you put headings on each of the subsections (for C, M, S, P, and I) that labelled the contents of each subsection.*

**Response**

 We will put headings on each of the subsections (for C, M, S, P, and I) that labelled the contents of each subsection.

**Comment**

*b) You are (quite appropriately) approaching AE detection as a screening test with 2 main steps. Step 1 involves evaluation of the initial triggers. Step 2 involves following up on positive initial triggers, to see if a validated AE emerges. This is done in the context of a general review by a trained clinical expert, who can (and sometimes does) detect AEs that fall outside of the trigger system.*

*It would be very useful if you separated the total time spent on CR review into (a) initial assessment of the triggers; versus follow-up analysis of the positive triggers.*

**Response**

 We are unclear of the changes required on this point.

**Comment**

*it would similarly be very useful if, in the 3rd column of Table 2 (labelled “Times associated with AEs, n (%)” you showed the proportion of positive triggers that yielded a confirmed AE related to/deriving from the initial positive trigger. The Table, as currently constructed, is quite confusing – in 10 instances (C03, C04, C08, C11, C12, C14, M02, M04, I01, I03) the count in 3rd column is larger than the count in 2nd column. Presumably, this is because you are not connecting the trigger to derivative AEs (but I can’t really tell, with any certainty).*

**Response**

 In the third column of table 2 we reported the number of times a trigger was found.

 In the fourth column the number of times in which an adverse event was related to that trigger.

 The title of the fourth column (Times associated, with AE)does not express this clearly.

 Therefore in the third column the triggers are indicated, while in the fourth column the adverse events are indicated. We have found some CRswith more than one adverse event. In the third column of table 2 we intended to report that the C03 trigger was found in 279 CRs examined. Instead, in the fourth column of Table 2 we wanted to indicate that in 438 adverse events the C03 trigger was present.

**Comment**

*The section entitled “Rates of AEs”, first paragraph, contains the sentence:*

*“It would appear that the percentage of clinical records with triggers increases with the increase of percentage of CRs examined, compared to patients discharged, while the percentage of CRs with AEs remains more stable.”*

*I can’t translate quite what that means. Please simplify and clarify. It may relate to the 2 upper lines in Figure 2, which both appear to be increasing over time. However, you supply no statistical analysis to show that trends over time in the 2 lines are statistically associated, and you offer no reasoning as to why such a relationship may have meaning.*

**Response**

 We mean that we did not find a correlation between the number of CRs examined in the period and the number of EAs observed in the same period. We will try to verify if the differences are statistically significant.

**Comment**

*Table 3 appears to identify high frequency triggers, then count the number of AEs of any sort that occur in CRs that had a high frequency trigger. It would be much more useful if you tracked each trigger to related or derivative AEs, rather than treating AEs generically.*

**Response**

 This would be very interesting to describe, but we do not know if it will be possible to provide this kind of detail due to editorial guidelines. It could be the subject of a new study to be published later.

**Comment**

*In the section labeled "Rates of AEs" you have a bulleted list. The last item in that list says:*

*"Intensive-care-related triggers have been identified 1,817 times with an AE in 1,924 (i.e., it is very common for triggers to identify more in the same patient)".*

*I think I get the gist of what you mean, but (a) this sentence needs more clarity; and (b) the main purpose of identifying AEs is to move towards intervention and prevention (better, safer care). In that circumstance, treating AEs as generic items is not very useful. It is necessary to list them by specific causes. In that framing, associating triggers (which are cause specific) with a total generic AE count is not very informative or useful. You might want to rethink your framing.*

**Response**

 This would also be interesting to describe. We have been required to be fit in with te word count and   to comply with the editorial guidelines. We will clarify this section

# Peer review: Reviewer response for version 1

**DOI:** 10.5256/f1000research.19713.r45400

**Published:** 2019-03-28

**Authors:** James M. Naessens

**Affiliations:** 1 Division of Health Care Policy and Research, Mayo Clinic, Rochester, MN, USA

This paper by Parrinello *et al.*, presents the experience of applying the Italian version of the Institute for Healthcare Improvement’s Global Trigger Tool (GTT) for screening for adverse events (AEs) to a random sample of medical records for hospital discharges from June 2015 to June 2018 for all 44 public hospitals in Sicily. The authors have done a nice job of describing their overall experience with the GTT and focused on the quantity and type of triggers which helped identify adverse events. There were a few issues where some further explanation would clarify the methods and results, and aid the reader in evaluating the contributions of this paper. In addition, there were a couple of minor editing points:

- In my first reading of the paper, I was confused by Table 2. It is not clear how a trigger could be associated with AEs more often than it is detected. This happened with C03, C04, C08, etc. Table 3 presents some of the same information, but labels the second column as AEs with triggers. To evaluate the effectiveness of GTT as a screening tool, it is useful to see both what percent of records with a specific trigger had an AE as well as how many AEs were associated with that trigger. The authors should clarify in **Methods** how they identified AEs (role of physician, why record without a trigger was reviewed) and clarify whether results they present are based on the count of AEs or the percent of records with AEs (or both). I’d recommend both, as in Table 1.        
- The first paragraph of the results states that 18,008 records were reviewed, but only 14,706 were analyzed. The authors should explain why the records were excluded, to show they did not bias the results.  A flow chart might help.
- They also state that 7 CRs had AEs without triggers. My understanding of the GTT method is that only records with triggers are reviewed for AEs. Please explain how these AEs were found.
- The first paragraph under “Rates of triggers” in the discussion, which should be moved to the **Results **section, refers to significant differences. The method of testing these statistics should be added to the “Statistical analysis” section of the **Methods.**

There were several typos or minor errors:

- P3, Methods, Review team: 71-person team (?).
- Table 1, lines 2 vs. 4 and lines 3 vs. 5: not clear what the differences are. How do we get different %’s?
- Figure 1: style of sensitivity line in graph does not match that of label.
- Table 5: Procedure complication has an asterisk, but no explanation.
- Figure 2: typo (EA) in 3 ^rd^ label.
- P9: surgery-elated (?).

 

 After reading the manuscript, it appears that there are two main questions that can be answered from the results:

- How does patient safety in Sicily, based on the GTT, compare to other published studies?
- Can we learn anything from the analysis of the triggers that can help us improve the review process?

The first question is addressed in the discussion (page 4). Adding a confidence interval to the reported rate from this study (6.6%) would clarify that these hospitals are as good or better than other reported rates.

 The most important conclusions I would draw from the trigger analysis is that few AEs were identified by isolated triggers and many isolated triggers are not associated with AEs. Although not highly useful for identifying AEs, some isolated triggers may be direct measures of “near misses”. I would restructure the results and discussion to emphasize these points.

 Overall, this is a good description of a broad-based standardized implementation of the Global Trigger Tool. The authors have presented suggestions for making improvements to the method.

Is the work clearly and accurately presented and does it cite the current literature?

Yes

If applicable, is the statistical analysis and its interpretation appropriate?

Partly

Are all the source data underlying the results available to ensure full reproducibility?

No source data required

Is the study design appropriate and is the work technically sound?

Partly

Are the conclusions drawn adequately supported by the results?

Partly

Are sufficient details of methods and analysis provided to allow replication by others?

Partly

Reviewer Expertise:

Health Services Research, Patient Safety and Quality of Care Measurement, Biostatistics

I confirm that I have read this submission and believe that I have an appropriate level of expertise to confirm that it is of an acceptable scientific standard, however I have significant reservations, as outlined above.

# Response

**Published:** 2019-04-03

**Authors:** vincenzo parrinello

**Affiliations:** U.O. Quality management and patients safety - Azienda Ospedaliero-Universitaria Catania, Italy, Italy

We appreciate the review by Professor Naessens of our paper, the accuracy of his observations and the relevance of his suggestions. We are honored that he considers our paper “ *a good description of a broad standardized implementation of the Global Trigger Tool*” and that our work “ *presented suggestions for improving the method.*”

We are aware that some of the points require further clarity, which may have been lost as we limited the word count.

**Comment**

*In my first reading of the paper, I was confused by Table 2. It is not clear how a trigger could be associated with AEs more often than it is detected. This happened with C03, C04, C08, etc. Table 3 presents some of the same information, but labels the second column as AEs with triggers. To evaluate the effectiveness of GTT as a screening tool, it is useful to see both what percent of records with a specific trigger had an AE as well as how many AEs were associated with that trigger. The authors should clarify in Methods how they identified AEs (role of physician, why record without a trigger was reviewed) and clarify whether results they present are based on the count of AEs or the percent of records with AEs (or both). I’d recommend both, as in Table 1*.

**Response**

**Table 2**

In the third column of this table we reported the number of times a trigger was found.

In the fourth column the number of times in which an adverse event was related to that trigger.

The title of the fourth column (Times associated, with AE)does not express this clearly.

Therefore in the third column the triggers are indicated, while in the fourth column the adverse events are indicated. We have found some CRswith more than one adverse event. In the third column of table 2 we intended to report that the C03 trigger was found in 279 CRs examined. Instead, in the fourth column of Table 2 we wanted to indicate that in 438 adverse events the C03 trigger was present.

**Table 3**

We have tried to elucidate this concept in Table 3.

This table lists the triggers that, in our experience, require more focus on. Finding one of the triggers on this list, linked to other triggers, could indicate the presence of more than one adverse event.

**Effectiveness of a GTT**

We agree that to evaluate the effectiveness of the GTT as a screening tool, it is useful to see both what percentage of CRs with a specific trigger have an AE and how many AEs have been associated with that trigger.  We have a table to explains this, but due to space constraints it was not included and we can add it if the editorial staff agree.

**Definitions and process**

Adverse events were identified based on the IHI protocol definition: " *unintended physical injury resulting from or contributed to medical care that requires additional monitoring, treatment or hospitalization, or that results in death.*" The physician reviewed the consensus with the two records and reached a final agreement on the type, number, and severity of events. The physician did not review the CRs, only the summary sheet in IT-platform.

**Table 1**

Table 1 shows the number of CRs examined, the number of CRs with triggers and the percentage of discharged patients. One line is missing which provides the number of discharged patients.

If the editorial staff allows, we can integrate Table 1 with the number of patients discharged and the number of AEs compared to the CRs examined and with triggers.

**Comment**

*The first paragraph of the results states that 18,008 records were reviewed, but only 14,706 were analyzed. The authors should explain why the records were excluded, to show they did not bias the results.  A flow chart might help.*

**Response**

From June 2015 to June 2018, 18.008 CRs were examined. In this study, we analyzed 14,706 CRs relating to patients discharged from 89 medicine, surgery, obstetrics and intensive care wards. 3.302 CRs concerned pediatrics and emergency department wards.

**Comment**

*They also state that 7 CRs had AEs without triggers. My understanding of the GTT method is that only records with triggers are reviewed for AEs. Please explain how these AEs were found.*

**Response**

Examination of the CRs was performed in accordance with the IHI protocol:

- Discharge codes, particularly infections, complications, or certain diagnoses
- Discharge summary
- Medications administration record
- Laboratory results
- Prescriber orders
- Operative records (operational report and record anesthesia, if applicable)
- Nursing notes
- Physician progress notes
- If time permits, any other areas of the record

In seven CRs the reading of the Discharge codes and of the Discharge summary has allowed us to identify directly the adverse events represented by two cases of invasive procedures complications, two cases of surgical complications, a case of hypoglycemia and a case of adverse reaction to the administration of a drug. Therefore the reviewers, having identified the adverse event, did not look for triggers.

**Comment**

The first paragraph under “Rates of triggers” in the discussion, which should be moved to the **Results **section, refers to significant differences. The method of testing these statistics should be added to the “Statistical analysis” section of the **Methods**

**Response**

The term "significantly" used in the first paragraph under "Rates of triggers" in the discussion, is misleading. We did not intend to claim that there was a statistically significant difference.

**Comment**

Style of sensitivity line in graph does not match that of label of Figure 1.

**Response**

if possible we will correct the figure.

**Comment**

*P3, Methods, Review team: 71-person team (?).*

**Response**

The total number of reviewers was 199 divided into 71 teams.

In some teams, medical reviewers  from another team were the supervisors.

**Comment**

*Table 1, lines 2 vs. 4 and lines 3 vs. 5: not clear what the differences are. How do we get different %’s?*

**Response**

In lines 2 and 4 of table 1 the number of CRs with triggers are shown.

In line 2, the % refers to the total number of patients discharged; in line 4 the % refers to CRs with triggers. For example, from June 2015 to June 2018, 1,571 medical cases with triggers were found in the medical departments, equal to 34.3% of all patients discharged in the same period (n = 4,527)  and 33.4% of all triggered CRs (n = 5,574). I think it would be clearer if we put a line with the number of patients discharged.

**Comment**

*Table 5: Procedure complication has an asterisk, but no explanation.*

**Response**

The explanation is Central Venous Catheter (CVC), Continuous Veno-Venous Hemofiltration (CVVH), Endoscopy, Orotracheal Intubation, Endoscopic Retrograde Cholangiopancreatography (ERCP).

If possible we will correct the table.

**Comment**

*Figure 2: typo (EA) in 3rd label.*

**Response**

Figure 2: typo (EA) in 3rd label. is a translation error (AE). In Italian it is EA,

**Comment**

*P9: surgery-elated (?).*

**Response**

it would be surgery-related.

**Comment**

*After reading the manuscript, it appears that there are two main questions that can be answered from the results: *

- *How does patient safety in Sicily, based on the GTT, compare to other published studies?*
- *Can we learn anything from the analysis of the triggers that can help us improve the review process?*

*The first question is addressed in the discussion (page 4). Adding a confidence interval to the reported rate from this study (6.6%) would clarify that these hospitals are as good or better than other reported rates.*

**Response**

Regarding the addition of a confidence interval at the rate reported by this study (6.6%) to clarify if these hospitals are equal or better than other reported rates, we think that the methodologies used in the various studies are too heterogeneous, protocols are often locally adapted to the local context, the populations studied are different, and the skills of the reviewers vary. For these reasons we decided not to explore this topic from a statistical point of view.

**Comment**

*The most important conclusions I would draw from the trigger analysis is that few AEs were identified by isolated triggers and many isolated triggers are not associated with AEs. Although not highly useful for identifying AEs, some isolated triggers may be direct measures of “near misses”. I would restructure the results and discussion to emphasize these points*

**Response**

We agree and we will emphasize this point.
